# Managing older adults’ fear of coronavirus disease: A new role for social work practice

**DOI:** 10.1177/1473325020973295

**Published:** 2021-03

**Authors:** Hamed Mortazavi

**Affiliations:** Gerontological Care Research Center, Department of Geriatric Nursing, School of Nursing and Midwifery, North Khorasan University of Medical Sciences, Bojnurd, Iran

**Keywords:** Older adults, community work, patients’ experiences, coronavirus

## Abstract

As the number of patients infected with the 2019 novel coronavirus disease (nCOVID-19) increases, the number of deaths has also been increasing. According to World Health Organization (WHO), as of 4 October 2020, 34,804,348 cases had tested positive for nCOVID-19 globally, which among them, 1,030,738 confirmed deaths had occurred, equivalent to a case-fatality rate of 2.96%. However, in comparison with global statistics, the incidence and mortality of the nCOVID-19 infection are higher in Iran. As reported by the National Committee on COVID-19 Epidemiology of Ministry of Health of Iran, the total number of patients with confirmed COVID-19 infection has reached 468,119, of which 26,746 have died, equivalent to a case-fatality rate of 5.71%. Currently, there is solid evidence that older adults are at a higher risk of severe disease following infection from COVID-19.

As the number of patients infected with the 2019 novel coronavirus disease (nCOVID-19) increases, the number of deaths has also been increasing. According to World Health Organization (WHO), as of 4 October 2020, 34,804,348 cases had tested positive for nCOVID-19 globally, which among them, 1,030,738 confirmed deaths had occurred, equivalent to a case-fatality rate of 2.96% (World Health Organization, 2020). However, in comparison with global statistics, the incidence and mortality of the nCOVID-19 infection are higher in Iran. As reported by the National Committee on COVID-19 Epidemiology of Ministry of Health of Iran, the total number of patients with confirmed COVID-19 infection has reached 468,119, of which 26,746 have died, equivalent to a case-fatality rate of 5.71% (National Committee on COVID-19 Epidemiology, 2020). Currently, there is solid evidence that older adults are at a higher risk of severe disease following infection from COVID-19. As reported by Centers for Disease Control and Prevention (CDC), 31–70% of older adults (65 years old and older) with confirmed COVID-19 require hospitalization, 6–31% of them require admission to intensive care unit and between 4 and 27% of them have died (approximately, 8 out of 10 deaths reported in the U.S. occur in adults 65 years old and older) (Centers for Disease Control and Prevention, 2020).

The rapid transmission of novel coronavirus and its associated high death rate could amplify fear and anxiety in the community, especially in the older adult with underlying lung disease, since most of the deaths caused by nCOVID-19 are related to the older adult patients (Li et al., 2020). For example, in an online survey of the general public in China, about 54% of respondents rated the psychological impact of the nCOVID-19 outbreak as moderate or severe and about 35% reported high levels of anxiety and stress (Wang et al., 2020). In addition, the health organizations constantly warn older people about the danger of the coronavirus disease, in particular those with a range of chronic underlying conditions such as cardiovascular diseases/hypertension and diabetes (Shahid et al., 2020). Accordingly, since older people are facing the most threats and challenges at this time, physical and psychological support for them is a necessity.

Some important issues make older adults psychologically vulnerable during the outbreak of nCOVID-19. Many of the older adults have high levels of functional dependency, behavioral and psychological problems such as anxiety and depression (Goldberg et al., 2012), which can worsen their fear about nCOVID-19 infection. In addition, many older adults have at least one chronic disease and therefore, physical frailty and long-term disabilities are common problems among older adults (Maresova et al., 2019; Mortazavi, 2018). Consequently, many of them require out-of-home nursing and medical care such as periodic visits by a physician (Tabatabaeichehr et al., 2018). However, at this time, they have to stay at home because of the spread of coronavirus and fear of infection. This can put their physical health at risk and make their concerns and fear worsen. Social media has also raised fear in this age group by repeatedly disseminating misinformation regarding the risk of death from nCOVID-19 infection in the older adults’ population. For example, in a qualitative study from Iran, the main reasons for misinformation distribution through the social network were cultural factors, demand pressure for information during the crisis, the easiness of information dissemination, and marketing incentives. This news has caused psychosocial problems in the general population, especially older adults, who need to be socially isolated (Bastani and Bahrami, 2020).

Fear and anxiety about the nCOVID-19 disease, which is often undetected and untreated, can be overwhelming and cause strong emotions in older adults. Anxiety can negatively affect their immune system outcomes, including inflammatory processes, wound healing, and responses to infectious agents and other immune challenges and weaken the body against disease (Segerstrom and Miller, 2004). Anxiety can sometimes lead to dysfunctional behaviors since uncertainty and magnification of risks are characteristics of anxious individuals (van Osch et al., 2014). For example, people may repeatedly send incorrect or unpleasant news about COVID-19 outbreak to each other, particularly via social spread and ignore correct messages and scientific instructions, which in turn increase individuals’ stress levels and anxiety surrounding this outbreak.

In response to this growing wave of concern in the community, it seems that healthcare providers, as well as social workers, have taken limited preventive measures to reduce fear and anxiety in this age group. A review of the recent research literature reveals that little attention has been paid to the psychological problems of older adults (Yang et al., 2020). Explicitly, no specific psychological and supportive interventions have been defined to support this age group to reduce fear and anxiety so far. Considering current restrictions on public transport, it is necessary for healthcare providers and social workers to develop and implement strategies to improve the health of older adults and reduce their fears and anxieties.

The important issue during the COVID-19 outbreak is that the provision of supportive and psychological interventions need to be changed from face-to-face to remote care. In this regard, previous studies have shown that health professions could play important roles in working with patients' families to provide home-based remote care and facilitate self-management of older adults (Hagedoorn et al., 2020). Remote care can be helpful at this time because innovative remote technologies have facilitated the development of services that allow older adults to stay at home (De Cola et al., 2020). In addition, providing remote care consultative services such as online mental health services and telephone consultation could be adopted to reduce their fears. Undoubtedly the correct use of online media can advance health-related knowledge such as information for specific conditions and disorders to relieving stress, and raising feelings of control and self-efficacy (Leist, 2013). In addition, during remote consultations, healthcare providers and social workers should emphasize that older people seek accurate information and obtain it from credible sources such as CDC, the World Health Organization, and the dedicated website of Medical Universities. Furthermore, preparing self-care training pamphlets related to their chronic diseases and sharing those pamphlets thorough social networks can be helpful. For example, the Ministry of Health of Iran and the universities of medical sciences publish instructions and recommendations related to the COVID-19 on their website on a daily basis to prevent misinformation dissemination among the public (http://corona.behdasht.gov.ir/). Moreover, to prevent nCOVID-19 infection, training proper personal hygiene through video, animation, and television programs is very important.

In Iran, the increasing prevalence of coronavirus has disrupted the normal daily life of the people and has caused anxiety and stress in the community, especially older adults. In this regard, several attempts have been done by the Iranian Scientific Association of Social Work to help and inform social work practice related to mental health in older adults during the coronavirus pandemic. This association has implemented a project called “Remote psychosocial support” to reduce the burden of stress caused by the critical outbreak of coronavirus by providing telephone counseling, remote supportive care, and referral counseling. This project was one of the widespread collaborative works done by social work practice in Iran. The first step in this project was to invite social work graduates and train them by prominent professors in the fields of medicine, psychology, and social work. In this project, trained social workers provide counseling to members of the community in special centers under the scientific supervision of a professor and refer suspected persons to hospital centers. Another activity of this association was publishing educational content on the association's website (www.swi.ir) on a daily basis about coronavirus disease, principles of self-care at work and home, principles of self-care of special groups and educational booklets and play at home for children. Establishing social health centers in high-risk areas, especially in the suburbs and high-traffic areas for education and awareness, as well as counseling to reduce stress in the community is another activity that was done in Iran. Increasing risk perception in community members was one of the measures taken continuously by all groups of medicine, psychology, and social work. All of these activities demonstrated the active roles of social workers along with other health disciplines to help people to reduce the spread of the Coronavirus and its consequences. However, there is still stress and anxiety among the people.

In general, designing and implementing creative and collaborative remote care for older adults, who are isolated at home due to fear and anxiety of getting infected with coronavirus, can be an important issue for both social workers and community nurses. In this regard, along with healthcare providers, social workers are in a good position to play a more active role to support the physical and mental health of older adults during the COVID-19 outbreak. However, research data are needed to develop evidence-based strategies to reduce adverse psychological impacts of the COVID-19 pandemic. Investigating the fear and anxiety of the home-dwelling older adults about infectious diseases and how to help them requires the multifaceted cooperation of healthcare providers and social activists such as social workers. However, due to the emerging nature of this virus and the lack of sufficient knowledge about it, there is a critical need for field research to explain how older adults encounter, manage and cope with this fear. A qualitative exploration of how older adults encounter with the fear of the COVID-19 outbreak can serve as a starting point for designing and implementing fear-reducing interventions. The following questions can guide the design of research-based interventions to expand the role of social workers in reducing coronavirus-related fear and anxiety. For example, what effect does coronavirus have on the daily lives of older adults? How do they overcome their fears? How do they manage their chronic and acute physical and mental problems during the outbreak of coronavirus? What is the experience of older adults who get infected with coronavirus? Answering these questions can be the key to designing interventions that make the role of the social work profession more remarkable in society than ever before.
